# Supplementary material for: Changes of Phytochemical Components (Urushiols, Polyphenols, Gallotannins) and Antioxidant Capacity during Fomitella fraxinea–Mediated Fermentation of Toxicodendron vernicifluum Bark
Source: Molecules. 2019 Feb 14;24(4):683. doi: 10.3390/molecules24040683 (PMC6412378; doi:10.3390/molecules24040683)
Supplement: Supplementary file 1 [file molecules-24-00683-s001.pdf]

Changes of Phytochemical Components (Urushiols, Polyphenols and Gallotannins) and Antioxidant Capacity during *Fomitella fraxinea*–mediated Fermentation of *Toxicodendron vernicifluum* Bark

Da-Ham Kim[1], Min-Ji Kim[1], Dae-Woon Kim[1], Gi-Yoon Kim[1], Jong-Kuk Kim[1], Han-Seok Choi[2], Young-Hoi Kim[1] and Myung-Kon Kim[1*]

[1] Department of Food Science and Biotechnology, Chonbuk National University, Jeonju 54896, Jeonbuk, Republic of Korea

[2] Department of Agriculture and Fisheries Processing, Korea National College of Agriculture and Fisheries, Jeonju 54874, Jeonbuk, Republic of Korea

***** **C**orrespondence: kmyuko@jbnu.ac.kr (M. K. K.); Tel.: +82-63-270-2551 (M. K. K.); +82-63-270-2572 (M. K. K.)

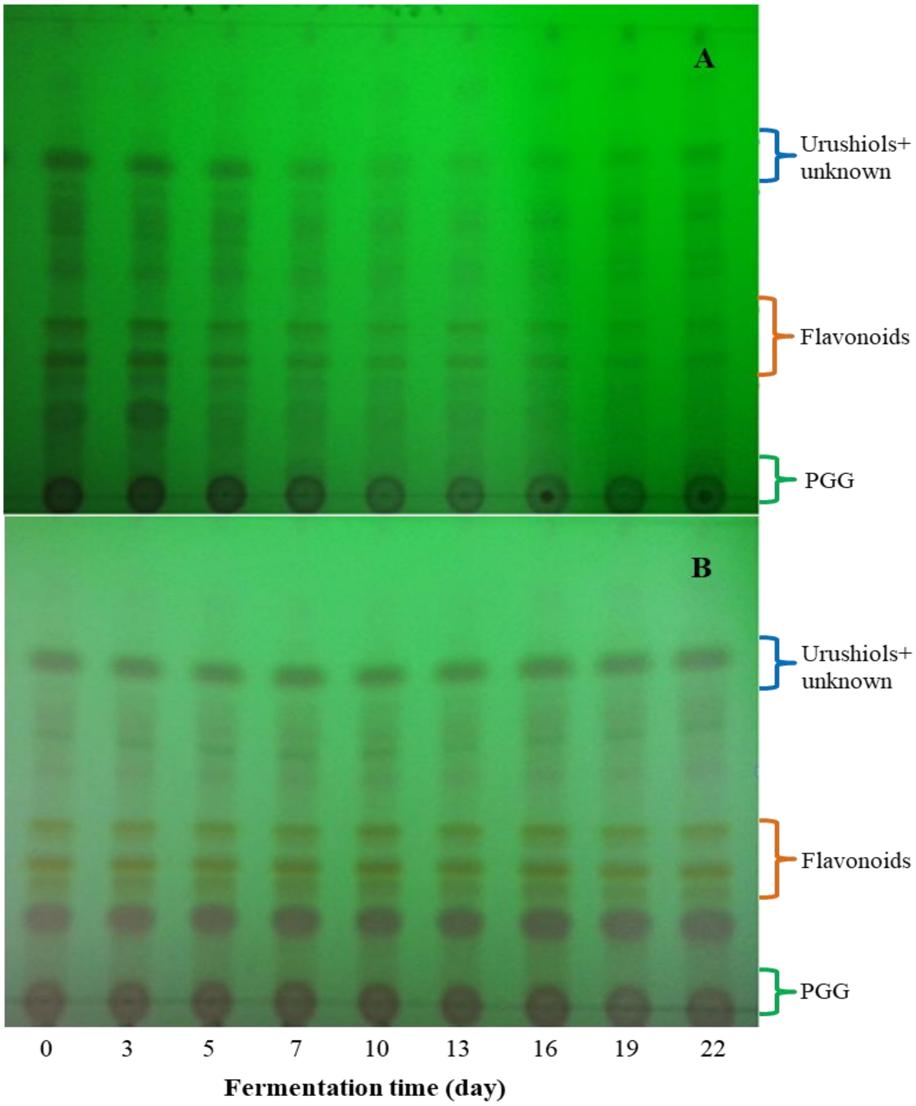

Figure S1. Profile comparison of thin layer chromatograpy (TLC) during 22 days fermentation of TVSB by *F. fraxinea*. **A**, fermentation during 22 days at 25 – 26 ℃ after *F. fraxine*a inoculation; **B**, fermentation during 22 days at 25 – 26 ℃ without *F. fraxinea* inoculation. TLC was performed on silica gel 60 F_{254} with chloroform–methanol-water (65:35:10, v/v/v, lower phase) as the developing solvent. The spots were detected at UV (254 nm).

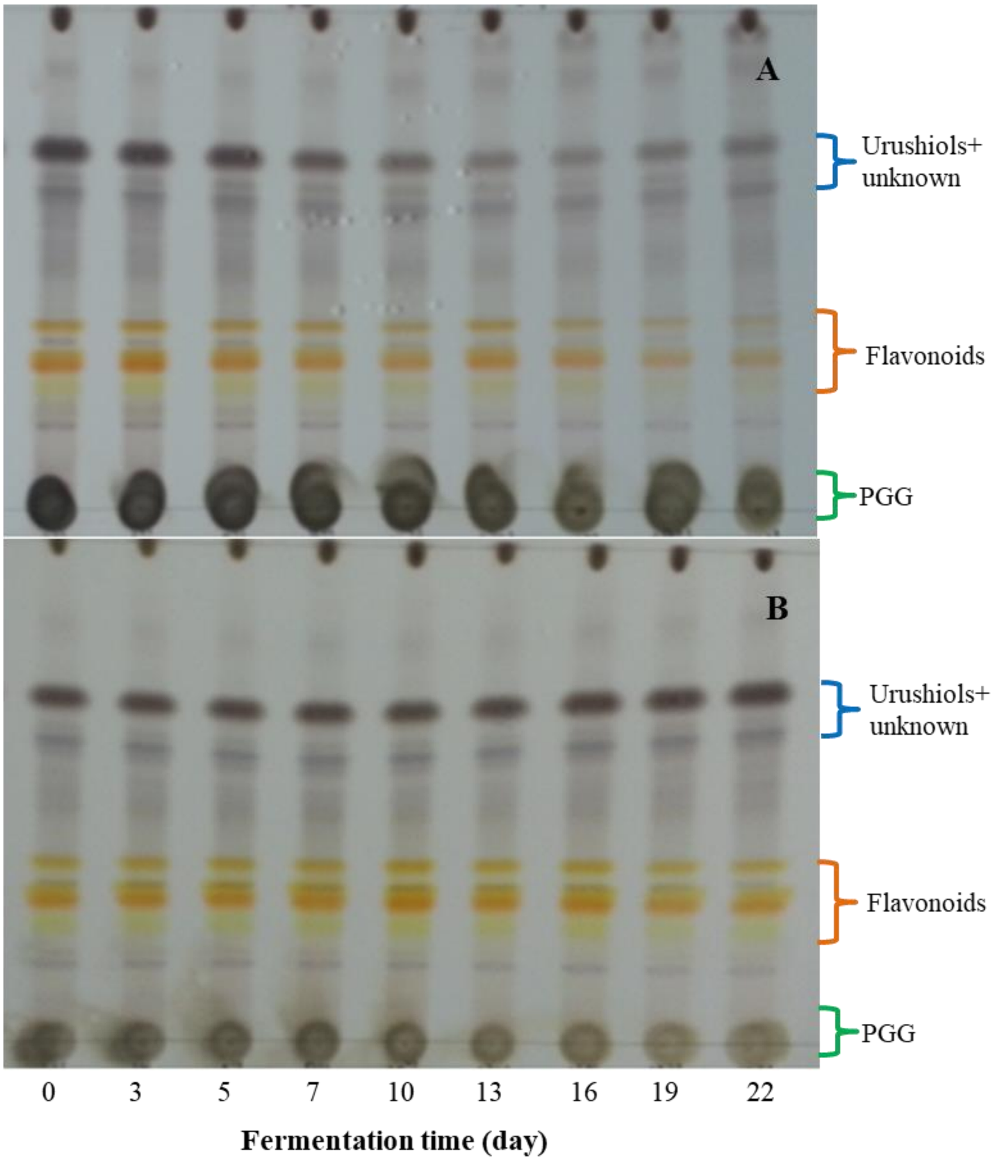

Figure S2. Profile comparison of thin layer chromatography (TLC) during 22 days fermentation of TVSB by *F. fraxinea*. **A**, fermentation during 22 days at 25 – 26 ℃ after *F. fraxine*a inoculation; **B**, fermentation during 22 days at 25 – 26 ℃ without *F. fraxinea* inoculation. TLC was performed on silica gel 60 F_{254} with chloroform–methanol–water (65:35:10, v/v/v, lower phase) as the developing solvent. The spots were detected by spraying 10% $CuSO_4$ in 8% sulfuric acid, followed by heating at 110 ℃ for 10 min.

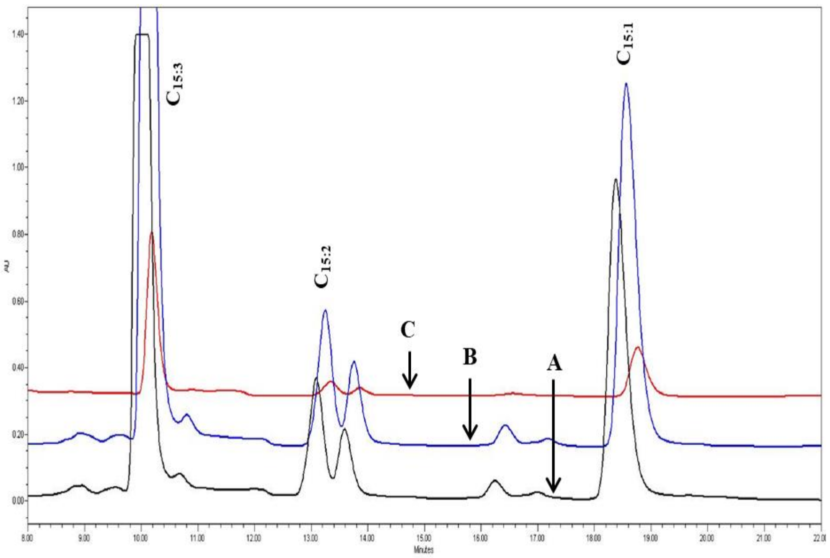

Figure S3. Profile comparison of HPLC of urushiols after 22 days fermentation of TVSB by *F. fraxinea*. **A**, 0 day (after sterilization at 121 ℃ for 30 min); **B**, after 22 days without *F. fraxinea* inoculation; **C**, after fermentation during 22 days after *F. fraxinea* inoculation.

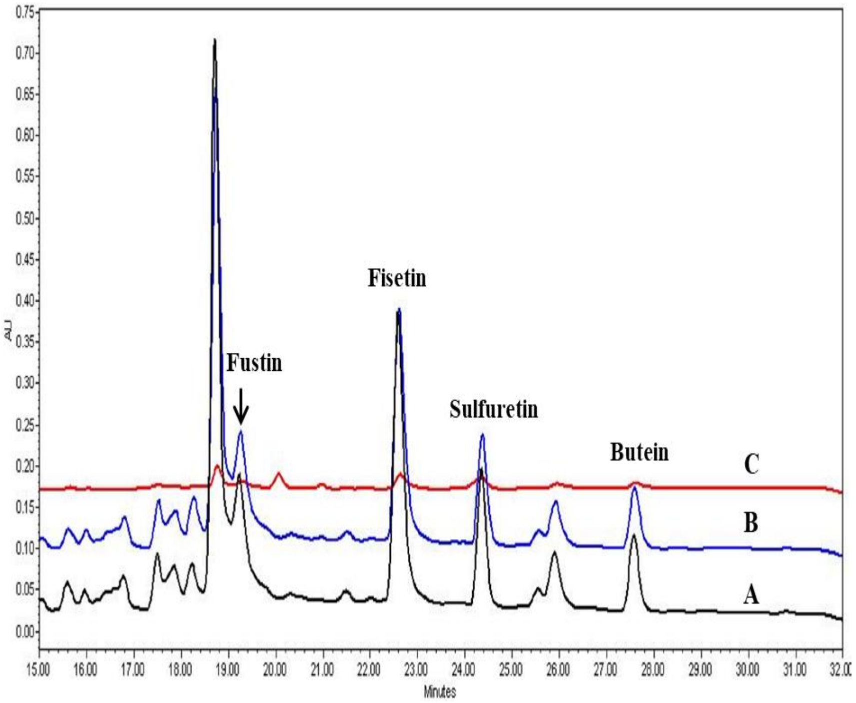

Figure S4. Profile comparison of HPLC of flavonoids after 22 days fermentation of TVSB by *F. fraxinea*. **A**, 0 day (after sterilization at 121 ℃ for 30 min); **B**, after 22 days without *F. fraxinea* inoculation; **C**, after fermentation during 22 days after *F. fraxinea* inoculation.
